# Regulation of ubiquitination in sepsis: from PAMP versus DAMP to peripheral inflammation and cell death

**DOI:** 10.3389/fimmu.2024.1513206

**Published:** 2024-12-10

**Authors:** Yueying Li, Jiongyan Yu, Zhiwen Zeng, Weixiong Lin

**Affiliations:** Department of Anesthesiology I, Meizhou People’s Hospital, Meizhou, Guangdong, China

**Keywords:** sepsis, peripheral inflammatory factors, ubiquitination, PAMP, DAMP, necrotic apoptosis

## Abstract

Sepsis (sepsis) is a systemic inflammatory response triggered by infection, and its pathologic features include overproduction of peripheral inflammatory factors (e.g., IL-1β, IL-6, TNF-α), which ultimately leads to cytokine storm and multiple organ dysfunction syndrome (MODS). Pathogen-associated molecular patterns (PAMP) and damage-associated molecular patterns (DAMP) induce strong immune responses and exacerbate inflammation by activating pattern recognition receptors (PRRs) in the host. Ubiquitination, as a key protein post-translational modification, dynamically regulates the activity of several inflammation-associated proteins (e.g., RIPK1, NLRP3) through the coordinated action of the E1, E2, and E3 enzymes, affects cell death pathways such as necroptosis and pyroptosis, and ultimately regulates the release of peripheral inflammatory factors. Deubiquitinating enzymes (DUBs), on the other hand, influence the intensity of the inflammatory response in sepsis by counter-regulating the ubiquitination process and balancing pro- and anti-inflammatory signals. This review focuses on how PAMP and DAMP activate inflammatory pathways via PRRs, and the central role of ubiquitination and deubiquitination in the development of sepsis, especially the mechanisms in regulating the secretion of peripheral inflammatory factors and cell death. By deeply dissecting the impact of the balance of ubiquitination and deubiquitination on inflammatory regulation, we further envision its potential as a therapeutic target in sepsis.

## 1 Introduction

**Figure 1:**
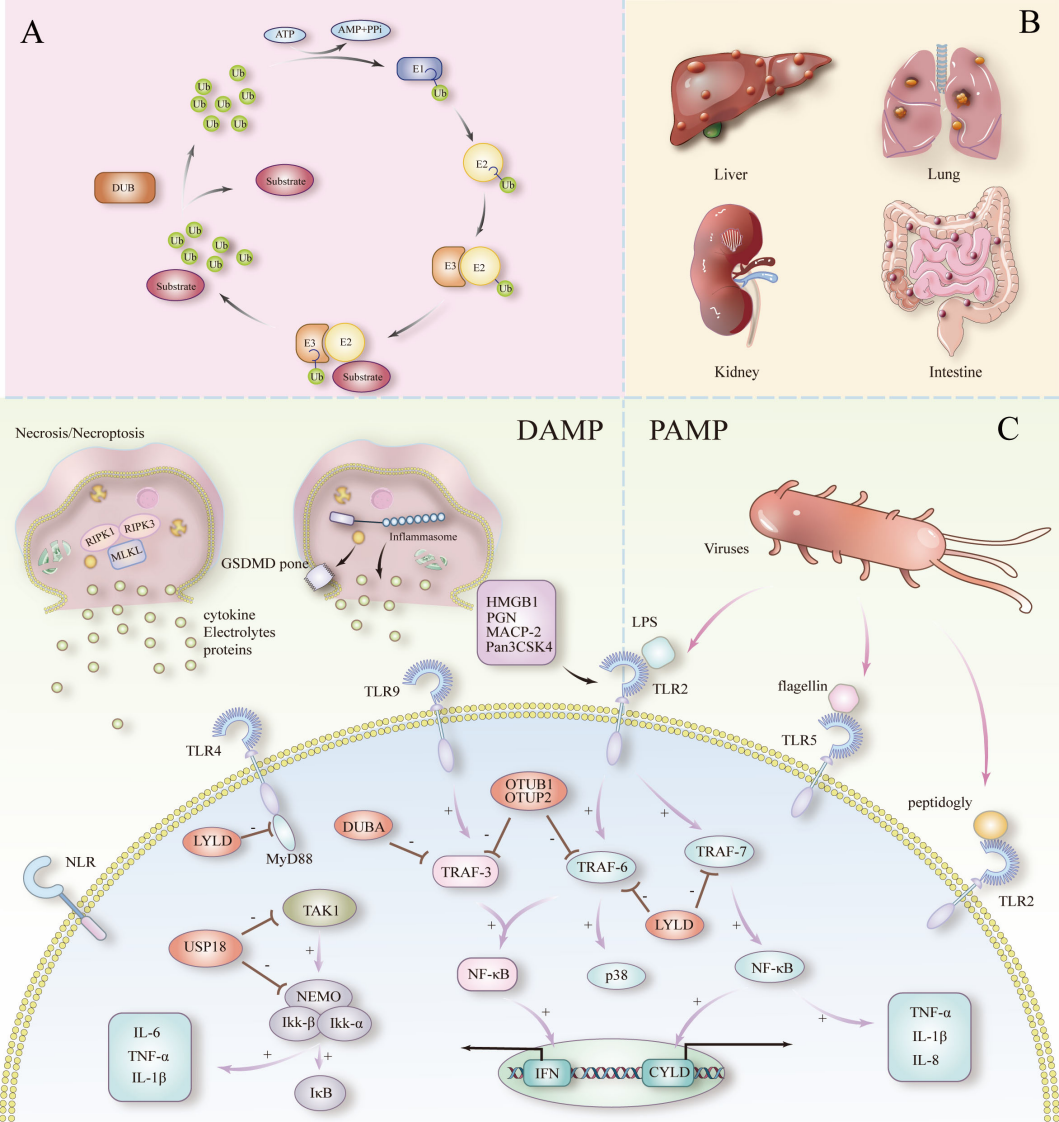
**(A)** Ubiquitination and deubiquitination processes. **(B)** Septicemia triggering multiple organ failure. **(C)** PAMPs and DAMPs to peripheral inflammation and cell death in sepsis.

Sepsis (sepsis) is a dysfunctional host response to infectiont, which is characterized by the abnormal secretion of peripheral inflammatory factors such as IL-1β, IL-6, and TNF-α (1–3). This condition is increasingly relevant in critically ill patients due to a rising incidence, which may reflect factors like an aging population and the prevalence of comorbidities (2, 4). These peripheral inflammatory factors act as powerful pro-inflammatory mediators, which not only drive local inflammatory responses, but also spread through the whole body, leading to the outbreak of cytokine storms, which ultimately lead to multi-organ dysfunction and failure (5) (**Figure 1B**). These peripheral inflammatory factors act as powerful pro-inflammatory mediators that not only drive localized inflammatory responses, but also spread systemically, leading to an outbreak of cytokine storms, which in turn triggers systemic inflammatory response syndrome (SIRS) and ultimately leads to multi-organ dysfunction and failure (5, 6).The persistent elevation of peripheral inflammatory factors is an important pathological feature in the development of sepsis, reflecting the state of immune imbalance (2, 6–8). In sub-Saharan Africa, the incidence of sepsis was reported to be 1,772 cases/100,000 people with a hospital mortality rate of 23.7% during 2013-2016 (9). The pathologic progression of this disease is triggered by pathogen-associated molecular patterns (PAMP) and damage-associated molecular patterns (DAMP), which induce an inflammatory response through the activation of host pattern-recognition receptors (PRRs), forming a pro-inflammatory cascade (10, 11).

Ubiquitination, a key post-translational modification of proteins, regulates the function and localization of target proteins through the synergistic action of E1, E2, and E3 enzymes, while deubiquitinating enzymes (DUBs) reverse this regulatory process by excising the ubiquitin chain (12, 13). Ubiquitination and deubiquitination dynamically modulate necroptosis by regulating inflammation-related proteins involved in apoptosis and pyroptosis. This regulation influences the release of peripheral inflammatory mediators, ultimately shaping the inflammatory response in sepsis patients. Both PAMPs and DAMPs activate pro-inflammatory signaling pathways via PRRs, while ubiquitination finely tunes the intensity and duration of this inflammatory response by modulating key proteins (e.g., NF-κB) within these signaling pathways. DUBs such as CYLD and A20 control the inflammatory response by removing the K63 of the RIPK1 chain ubiquitin and inhibit the overactivation of downstream pro-inflammatory signaling to prevent uncontrolled inflammation, revealing that the dynamic balance between ubiquitination and deubiquitination is critical for the regulation of inflammation in sepsis (14). This process holds significant clinical promise as a potential therapeutic target.

The aim of this review is to delve into the multilevel regulatory mechanisms of ubiquitination and deubiquitination in sepsis, focusing on the process by which PAMP and DAMP trigger inflammatory responses through activation of PRRs, and how they regulate the activity and function of key inflammatory proteins (e.g., RIPK1 and NF-κB) via the ubiquitination pathway. We will elaborate how these regulatory mechanisms affect cell death and immune cell activation (15–17).

## 2 The ubiquitination system and its key role in sepsis

Ubiquitin is a small protein consisting of 76 amino acids and is present in all tissues of eukaryotes (18). With only three amino acid differences between mammals, yeast and plants, it shows significant evolutionary conservation. It acts as a modifier by covalently attaching to cellular proteins via an enzymatic cascade, which involves three classes of enzymes called E1 (activation), E2 (binding), and E3 (ligation) (19). The process of ubiquitin labeling of proteins is called ubiquitination, and the process involves three major steps: first, E1 activating enzymes activate the ubiquitin molecule; then, E2 binding enzymes receive and transfer the ubiquitin; and finally, E3 ligase covalently attaches ubiquitin to the lysine residues of the target protein. Ubiquitination is one of the most versatile cellular regulatory mechanisms known to be used to control cell death and immune-inflammatory responses (**Figure 1A**). In sepsis, E3 ligases such as TRAF6 amplify the inflammatory response by activating the NF-κB and MAPK signaling pathways through K63 chain ubiquitination. RING-type and HECT-type E3 ligases play different roles in the recognition and modification of different inflammation-associated proteins (20, 21). Dysregulation of ubiquitination often results in a dysfunctional inflammatory response, exacerbating inflammation in sepsis. Out of control, exacerbating the cytokine storm in sepsis.

The process of ubiquitination is reversible. Deubiquitination is the opposite process of ubiquitination, where mono- or polyubiquitin chains are removed from a modified protein to terminate the ubiquitination function of the protein. In simple terms, the degradation of ubiquitinated proteins is prevented and their stability is maintained (14). Currently DUBs number about 100 in humans and are classified into six structurally distinct DUB families including UCHs (ubiquitin carboxyl-terminal hydrolases) of human USPs (ubiquitin-specific proteases), OTUs (ovarian tumor proteases), MJDs (Machado- Josephin’s disease protein structural domain proteases), MINDYs (motifs with containing ubiquitin (MIU) and JAMMs (Zn-JAB1/MPN/MOV34 structural domain proteases) (22–25). The first five belong to the cysteine proteases (26). Among them, the USP sub family is the most diverse member of the DUB family (27, 28). For example, DUBs in the nucleus (e.g., USP10) regulate gene transcription and DNA repair, whereas DUBs located in the cytoplasm (e.g., CYLD and A20) regulate inflammation-associated signaling pathways, such as NF-κB and MAPK. In sepsis, cytoplasmic DUBs (CYLD and A20) ubiquitylize the K63 chain of key proteins, such as RIPK1 and NEMO, by removing the inhibit overactivation of NF-κB and MAPK signaling, thereby reducing cytokine release and inflammatory spread. This distribution and functional specificity suggests that DUBs finely regulate immune responses in different cellular regions, with potential clinical implications for the maintenance of immune homeostasis in sepsis patients.

## 3 DAMP and PAMP production and their role in death: mechanisms of ubiquitination regulation

DAMP (damage-associated molecular pattern) and PAMP (pathogen-associated molecular pattern) are key factors in triggering inflammatory responses and cell death. DAMP is released by damaged or dead cells, whereas PAMP originates from exogenous pathogens. Both initiate a strong innate immune response through activation of host pattern-recognition receptors (PRRs), such as Toll-like receptors (TLRs) and nucleotide-binding oligomerized structural domain-like receptors (NLRs), initiating a strong innate immune response. This activation not only leads to localized inflammation, but also induces SIRS, exacerbating the cytokine storm and ultimately leading to multi-organ damage. In sepsis, the persistence of DAMP and PAMP contributes to an uncontrolled inflammatory response, in which ubiquitination and deubiquitination mechanisms finely regulate inflammatory intensity and cell survival by modulating key signaling molecules (e.g., RIPK1, NF-κB).

### 3.1 DAMP production and its role in inflammation

DAMP are endogenous molecules released by damaged or dead cells during the process of death. These molecules include HMGB1 (high mobility group protein B1), ATP, uric acid crystals, heat shock proteins, etc. (10, 29). DAMP plays a key inflammatory-activating role in sepsis, by binding to specific PRRs (e.g., TLR and NLRs), triggering massive activation of immune cells (e.g., macrophages, neutrophils) and secretion of peripheral inflammatory factors (10, 11, 30) (**Figure 1C**). This process allows the inflammatory reaction to spread rapidly, forming a vicious cycle. Particularly in sepsis, the sustained release of DAMP is closely associated with extensive tissue damage, and this vicious cycle ultimately triggers a cytokine storm that results in multi-organ failure.

### 3.2 PAMP production and its role in inflammation

PAMP are carried by exogenous pathogens, including lipopolysaccharide (LPS) and peptidoglycan from bacteria, double-stranded RNA from viruses, and β-glucan from fungi (31). These molecules activate downstream signaling pathways such as NF-κB, MAPK, etc., by binding to receptors such as TLR, NLR, and other receptors etc., triggering a strong innate immune response. In sepsis, pathogen mass multiplication and sustained PAMP stimulation keep the host’s immune system in a highly activated state, leading to the overproduction of peripheral inflammatory factors and their subsequent systemic inflammatory response.

### 3.3 Regulatory effects of ubiquitination on key proteins and cell death

In sepsis, both DAMP and PAMP are recognized by PRRs (e.g., TLR and NLR), leading to activation of inflammatory pathways and release of mediators. These mediators amplify the inflammatory response through feedback mechanisms, often leading to SIRS (32). However, the regulation of the inflammatory response does not only depend on these exogenous stimuli, but is also finely controlled by intracellular protein regulatory mechanisms, especially the process of ubiquitination. Ubiquitination, as a post-translational modification of proteins, dynamically regulates the function of several key proteins, which in turn affects the intensity and duration of the inflammatory response. By regulating the activity of inflammation-associated proteins such as RIPK1 (receptor-interacting protein kinase 1) and NLRP3, ubiquitination plays a key role in cell death and the release of peripheral inflammatory factors (33, 34).

#### 3.3.1 Effect of ubiquitination on necroptosis

Necroptosis, also known as programmed cell death, is a regulated, cell death mechanism dependent on the activation of RIPK1 (receptor-interacting protein kinase 1), RIPK3 and MLKL (mixed-spectrum kinase structural domain-like proteins) mediating cell death, which is characterized by a loss of cellular membrane integrity leading to the release of cellular contents into the extracellular matrix, which triggers an intense inflammatory response (35, 36). Ubiquitination controls the onset of necrotic apoptosis by regulating the activity of a series of key proteins, including RIPK1, RIPK3 and MLKL.

Ubiquitination of the K63 chain of RIPK1 by the E3 ubiquitin ligases IAPs activates pro-inflammatory downstream activity via NFKB (37). However, when RIPK1 is dysfunctional in its ubiquitination or de-ubiquitinated by CYLD, RIPK1 forms a complex with RIPK3 and further activates downstream MLKL, which induces necrotic apoptosis, leading to DAMP release, exacerbating the inflammatory response (38). In addition, deubiquitination of RIPK3 by CYLD increases its stability, making it more susceptible to forming a complex with RIPK1 and activating MLKL.

Ubiquitination of MLKL regulates its activity and membrane localization, and unubiquitinated MLKL binds more readily to RIPK3 and receives phosphorylation, resulting in oligomerization and translocation to the plasma membrane (39). Oligomerization of phosphorylated MLKL has been reported in the presence of highly phosphorylated inositol phosphates (IP6), resulting in the formation of necrosomes. translocation of MLKL oligomers in plasma membranes to phosphatidylinositol phosphate (PIP)-rich plaques and form macropores. Eventually, MLKL pores lead to necroptosis by allowing ion influx, cell swelling and membrane cleavage leading to uncontrollable release of intracellular substances (e.g. HMGB1, ATP, etc.) (40).

#### 3.3.2 Effect of ubiquitination on pyroptosis

Pyroptosis is usually achieved in sepsis through both classical and nonclassical pathways. Both pathways involve activation of inflammatory vesicles and the action of Gasdermin family proteins. The classical pathway involves activation of caspase-1 by inflammatory vesicles (e.g., NLRP3), which triggers cleavage of GSDMD; the nonclassical pathway involves activation of caspase-4, caspase-5, or caspase-11 by intracellular LPS, which directly cleaves GSDMD (41–43).

In the classical pyroptosis pathway, the K63 chain of NLRP3 is ubiquitinated by the E3 ubiquitin ligase TRIM31, which in turn inhibits the over-assembly of inflammatory vesicles, thus limiting the excessive occurrence of pyroptosis (44). However, in sepsis, dysregulation of ubiquitination (e.g., enhancement of the deubiquitinating enzyme BRCC3) increases the activity of NLRP3, which leads to the excessive activation of inflammatory vesicles, thereby exacerbating cellular pyroptosis and further releasing large amounts of pro-inflammatory cytokines (e.g., IL-1β and IL-18) (45).

In non-classical cleavage pathways, ubiquitination affects cellular responses to endogenous and exogenous stimuli by modulating the activity of these caspases. The current study found that the E3 ligase NEDD4 ubiquitinates modified caspase-11 (46, 47).

The role of pyroptosis in sepsis is twofold. In the early stages of infection, moderate pyroptosis helps the immune system to recognize and clear pathogens. However, in the advanced stages of sepsis, hyperactivation of pyroptosis and sustained release of peripheral inflammatory factors leads to a cytokine storm that further exacerbates systemic inflammatory responses and organ failure.

## 4 Inflammatory signaling activated by DAMP and PAMP via PRRs

Pattern recognition receptors (PRRs) are a central part of the innate immune system. PRRs, TLRs and NLRs, are the main sensing receptors in the initiation phase of sepsis (**Figure 1C**). Among them, TLRs are the most studied and relevant PRRs associated with cytokine storm and sepsis.

Toll-like receptor 4 (TLR4) is triggered by LPSand is the first mammalian paradigm for innate immune signaling (48). TLRs expressed at the plasma membrane (TLR1, 2, 4, 5, and 6) recognize a wide range of lipid- and protein-like ligands in the extracellular environment (49, 50). In particular, after recognizing LPS, TLR4 activates the NF-κB and MAPK signaling pathways, initiating a pro-inflammatory response that further triggers the sepsis-associated inflammatory cascade. It has been shown that TLR4 is particularly associated with sepsis, and TLR4-deficient mice exhibit reduced responsiveness in the face of LPS from Gram-negative bacteria. Further evidence for a central role of TLR4 in the development of sepsis (51).

In patients with sepsis, changes in the expression of TLR2 and TLR4 are more active on neutrophils than on monocytes. The expression of Toll-like receptor signaling genes in monocytes is reduced in more severe disease. In contrast, in the clinical phase of sepsis, neutrophil expression of these genes is upregulated (52). Like TLR4, TLR2 responds to the internalization of bacterial IL-12 (53).

The NLR is present in the cell and recognizes several types of ligands, including bacterial cell wall components, toxins, and host-derived molecules (e.g., ATP, uric acid, and damaged cell membranes), which in turn trigger the activation of different biological pathways. This ligand specificity stems from its NH2-terminal effector structural domain (54).

## 5 Deubiquitination and peripheral inflammatory factor regulation in sepsis

In the initial stage of sepsis, an intense inflammatory response is ignited, leading to a surge of large amounts of inflammatory factors (e.g., IL-1β, TNF-α), which in turn creates a cytokine storm. When local inflammation cannot be controlled, inflammation can spread throughout the body, leading to SIRS. As inflammation persists, immune function becomes progressively impaired and immunosuppression occurs, leading to viral reactivation, secondary infections, and ultimately increasing the risk of death in patients.

### 5.1 Type I IFN

Type I interferons are produced by a variety of cells (macrophages, conventional dendritic cells (cDCs), and inflammatory monocytes in response to activation of cell-surface and intracellular pattern-recognition receptors, in particular the TLR family (55). The cytoplasmic structural domains of all TLRs share a high degree of similarity with those of the IL-1 receptor type I, and together they assist in host defense against infection. Closely associated with innate immune response (56–58). In the absence of IL-1R1 signaling, in contrast to the level of ubiquitination of TRAF3, its degradative ubiquitination was unaffected because of the upregulation of DUBA (deubiquitinating enzyme A) at this time (59).

DUBA (deubiquitinating enzyme A) selectively cleaves the K63-linked ubiquitin chain from TRAF3 (60). DUBA short interfering RNA enhances TLR9-dependent type I IFN responses (59). Mice lacking IL-1RI signaling fail to produce protective type I IFN responses after administration of TLR9 ligand (CpG), suggesting that IL-1 signaling modulation of DUBA expression attenuates TLR9-mediated pro-inflammatory responses (59). Mice lacking IL-1RI signaling fail to produce a protective type I IFN response after administration of TLR9 ligand (CpG), suggesting that regulation of DUBA expression by IL-1 signaling attenuates TLR9-mediated proinflammatory responses.

In virus-induced sepsis, the virus triggers a strong inflammatory response by activating the host’s immune system, where overactivation of the type I interferon pathway and the NF-κB signaling pathway often leads to SIRS and cytokine storm (61, 62). Sustained high levels of pro-inflammatory signaling can contribute to the immune system imbalance, which in turn develops into sepsis (62, 63).

OTUB1 and OTUB2 of the OTU family specifically remove K63 chain ubiquitin on TRAF3 and TRAF6 to negatively regulate the virus-triggered type I interferon pathway. Overexpression of OTUB1 and OTUB2 inhibits the activation of IRF3 and NF-κB, which in turn attenuates the transcription of the IFNB1 gene and the cellular antiviral response (64). This deubiquitination suggests that OTUB1 and OTUB2 play a negative regulatory role in response to viral infections to prevent damage to the host from an over-activated inflammatory response.

### 5.2 IL family and TNF-α

The pro-inflammatory cytokines of the IL-1 family include IL-1α, IL-1β, IL-18, and IL-36, and the structure of the corresponding bound receptor consists of an extracellular Ig structural domain and an intracellular TIR structural domain (65).This process involves myeloid differentiation major response protein 88 (MyD88) and IL-1 receptor associated kinase 4 (IRAK4) recruitment, and activation of NF-κB and MAPK (66, 67). IL-8 (CXCL8) belongs to the chemokine family and is mainly involved in neutrophil recruitment (68, 69). TNF-α (tumor necrosis factor α or cachectin) belongs to the pro-inflammatory cytokine family and is secreted by a variety of immune cells (macrophages, monocytes, neutrophils) (70, 71).

Multiple deubiquitinating enzymes have been shown to be involved in TIR-mediated downstream signaling activation, thereby indirectly affecting the release of peripheral inflammatory factors. As the most studied DUB family member among USPs, CYLD (oncogene cylindromatosis) is induced by Gram-negative and Gram-positive bacterial pathogens or their products (72–74). Meanwhile, CYLD acts as a negative regulator in TLR2 signaling. When TLR2 ligands (e.g., peptidoglycan PGN, MALP-2, and Pam3CSK4) bind to TLR2, TRAF6 and TRAF7 are activated, initiating the downstream NF-κB and p38 pathways, which in turn promotes the release of TNF-α, IL-1β, and IL-8 (75). In the meantime, virtually all microbial products are also activated via the TLR ligands induced IL-1β production (76, 77). During this process, the transcriptional level of CYLD gradually increased (75).There was a negative regulatory effect of CYLD on TRAF6 and TRAF7, which inhibited the over-activation of these pro-inflammatory pathways by removing the ubiquitylation of K63 chain on TRAF6 and TRAF7. Overactivation of these pro-inflammatory pathways, creating a negative feedback mechanism to prevent deleterious inflammatory responses. Absence or deficiency of CYLD results in uncontrolled inflammatory responses that significantly exacerbate the severity of sepsis (75).

Recent studies have further revealed the negative regulatory role of CYLD in the MyD88-mediated TLR signaling pathway. It is well known that key receptors involved in sepsis initiation include toll-like receptors (TLRs) (78). The gram-negative bacterium NTHi (non-typeable Haemophilus influenzae), an infectious trigger of sepsis, generally initiates the host immune response through the TLRs signaling pathway (79–81). MyD88 is an adaptor molecule for the majority of TLRs (TLR2, TLR4) and IL-1R signaling (82, 83). It was demonstrated that CYLD removes the K63-connected ubiquitin chain by directly interacting with MyD88, specifically the ubiquitin modification located at lysine 231, thereby negatively regulating NTHi-induced MyD88-mediated signaling. This deubiquitination effectively inhibited NTHi-triggered pro-inflammatory cytokine production and prevented excessive inflammatory responses (84). In addition, mice were able to generate strong antibody responses to T-cell-dependent antigens, despite the lack of MyD88 and TRIF, two key components of TLR signaling, and the lack of a TLR ligand response (85).

In addition, a deubiquitinating enzyme, USP18, affects TNF-α and IL-1β levels. USP18, an interferon-inducible gene, is significantly up-regulated in TLR ligand-stimulated human monocytes and macrophages. LPS combined with TLR revealed increased phosphorylation of IKK, accelerated degradation of IκB, and higher levels of TNF-α, IL-6, and IL-1β mRNA expression in THP-1-derived macrophage species transfected with USP18-specific siRNAs (86).

## 6 Conclusions and perspectives

DAMPs released by necrotic apoptosis amplify the immune cell response to pathogens, while focal death exacerbates the cytokine storm in sepsis by releasing proinflammatory factors (e.g., IL-1β, IL-18). Consider the role of ubiquitination and deubiquitination at various stages of sepsis and control this process to prevent necrotic apoptosis and pyroptosis. Since ubiquitination and deubiquitination are independent yet interrelated processes, identification of specific deubiquitinating enzymes (DUB) corresponding to specific ubiquitinases may provide another effective strategy for regulating ubiquitination.

Multiple deubiquitinating enzymes are negative regulators of TLR signaling, but how they accomplish their job of inhibiting TLR overactivation and the associated cytokine storm has yet to be further explored. Sustained exposure to PAMPs (e.g., LPS released by bacteria) and DAMPs (e.g., molecules released by dying cells and damaged tissues of the host) leads to excessive and sustained activation of the TLR signaling pathway. With excessive activation of the TLR signaling pathway, cytokines such as TNF-α, IL-1β, and IL-6 are released in large quantities, ultimately triggering a cytokine storm. This cytokine storm drives the development of MODS by triggering a systemic inflammatory response, which in turn leads to organ damage and high mortality in sepsis patients. Therefore, in-depth studies on the regulatory mechanisms of TLR signaling are needed, especially on how to inhibit excessive cytokine storms by modulating the negative feedback or deubiquitination pathways of TLR signaling.
